# No more time to stay ‘single’ in the detection of *Anisakis pegreffii, A. simplex* (s. s.) and hybridization events between them: a multi-marker nuclear genotyping approach

**DOI:** 10.1017/S0031182016000330

**Published:** 2016-04-05

**Authors:** S. MATTIUCCI, V. ACERRA, M. PAOLETTI, P. CIPRIANI, A. LEVSEN, S. C. WEBB, D. CANESTRELLI, G. NASCETTI

**Affiliations:** 1 Department of Public Health and Infectious Diseases, Section of Parasitology, ‘Sapienza -University of Rome’, P.le Aldo Moro, 5 00185 Rome, Italy; 2 Department of Ecological and Biological Sciences, Tuscia University, Viale dell'Università s/n 01100 Viterbo, Italy; 3 National Institute of Nutrition and Seafood Research (NIFES), Strandgaten 229, N-5004 Bergen, Norway; 4 Cawthron Institute, Private Bag 2, Nelson 7042, New Zealand

**Keywords:** *Anisakis pegreffii*, *A. simplex* (s. s.), multi-marker genotyping approach, elongation factor 1 alpha1 nDNA (EF1 *α*-1 nDNA region), contemporary hybridization

## Abstract

A multi-marker nuclear genotyping approach was performed on larval and adult specimens of *Anisakis* spp. (*N* = 689) collected from fish and cetaceans in allopatric and sympatric areas of the two species *Anisakis pegreffii* and *Anisakis simplex* (s. s.), in order to: (1) identify specimens belonging to the parental taxa by using nuclear markers (allozymes loci) and sequence analysis of a new diagnostic nuclear DNA locus (i.e. partial sequence of the EF1 *α*−1 *n*DNA region) and (2) recognize hybrid categories. According to the Bayesian clustering algorithms, based on those markers, most of the individuals (*N* = 678) were identified as the parental species [i.e. *A. pegreffii* or *A. simplex* (s. s.)], whereas a smaller portion (*N* = 11) were recognized as F_1_ hybrids. Discordant results were obtained when using the polymerase chain reaction–restriction fragment length polymorphisms (PCR–RFLPs) of the internal transcribed spacer (ITS) ribosomal DNA (rDNA) on the same specimens, which indicated the occurrence of a large number of ‘hybrids’ both in sympatry and allopatry. These findings raise the question of possible misidentification of specimens belonging to the two parental *Anisakis* and their hybrid categories derived from the application of that single marker (i.e. PCR–RFLPs analysis of the ITS of rDNA). Finally, Bayesian clustering, using allozymes and EF1 *α*−1 *n*DNA markers, has demonstrated that hybridization between *A. pegreffii* and *A. simplex* (s. s.) is a contemporary phenomenon in sympatric areas, while no introgressive hybridization takes place between the two species.

## INTRODUCTION

Ecological and epidemiological studies require that actual parasite species can be accurately defined (Criscione *et al*. 2005). On the other hand, species delimitation, the process of identifying and delineating distinct taxonomic entities, remains among the most challenging tasks in the study of parasite biodiversity. Cryptic species of parasites may occur when one or more established characters (often morphology) for defining species limits are uninformative and/or incongruent (Pérez-Ponce de León and Nadler, 2010; Nadler and Pérez-Ponce de León, 2011). Thus, molecular methodologies provide a valuable source of additional markers in such cases of heterogeneity and fill the gap in delimiting populations and species of parasites. This also concerns the application of genetic markers in anisakid nematodes, demonstrating reproductive isolation in sympatry and allopatry, and in giving estimates of gene flow between them (Mattiucci and Nascetti, 2008). In other words, molecular markers, particularly nuclear ones, provide evidence for the existence of biological species (BSC, *sensu* Mayr and Ashlock, 1969). Despite the fact that in recent years the BSC concept has been supported by the Phylogenetic species concept (PSC, *sensu* Avise and Wollenberg, 1997), under which “*a species has been defined as a monophyletic group composed of a cluster of individual organisms within which there is a parental pattern of ancestry and descent*”, it has been suggested how principles of ‘genealogies concordance’ might be employed to combine elements of the PSC and the BSC (Avise and Wollenberg, 1997). Furthermore, rather than concentrating on a single character type (e.g. morphological or DNA sequence variation), a wide range of characters (including not only molecular/genetic data but also morphology, ecology, geography, pathogenicity, host specificity/host range, etc.) are integral to delimiting parasite species and assessing species boundaries.

It has been frequently reported that sympatric populations of closely related parasite species might interbreed (Agatsuma *et al*. 2000; Steinauer *et al.* 2008; Detwiler and Criscione, 2010), including parasitic nematodes (Anderson, 2001; Criscione *et al.* 2007; Dunams-Morel *et al.* 2012). Hybridization, through interspecific crossing between closely related species, could have major evolutionary consequences for species and populations by either promoting or preventing divergence, depending by the viability and reproductive abilities of the hybrids in producing backcrosses with parental species. Adaptive traits could be also acquired through hybridization, resulting in increased or lower fitness in hybrid individuals (Criscione *et al.* 2007; Gilabert and Wasmuth, 2013). Hybridization and introgression could lead to some phenotypic changes of pathogens, including parasites, such as the invasion of new hosts, new geographical areas and site of infection (Criscione *et al.* 2007; Detwiler and Criscione, 2010). Therefore, the accurate detection and characterization of hybridization is important both in basic and applied biology, and not only in free-living species but also in parasitic animals. Again, molecular/genetic markers, especially the nuclear ones, are making these analyses accessible *via* a large number of tested individuals.

However, hybridization, introgression and the retention of ancestral polymorphism in closely related parasitic taxa could generate patterns of genetic variation which complicate their disclosure, when choosing inappropriate genetic/molecular markers and/or when the detection of hybridization is inferred by genetic markers obtained from the analysis of a single locus. A common approach to identifying hybrids is the use of parental taxa specific diagnostic markers at nuclear loci, such as allozymes, which are fixed in the parental species over a large sample size from various hosts and geographical areas; mixed (heterozygote) banding patterns of offspring individuals, indicating natural hybridization can be detected. Discordance between nuclear and mitochondrial datasets has also been used to distinguish putative hybrids between closely related species (Okamoto *et al.* 2010). The availability of highly polymorphic genetic markers and Bayesian clustering methods, such as STRUCTURE (Pritchard *et al.* 2000) and NEWHYBRIDS (Anderson and Thompson, 2002), has also improved our knowledge of hybridization events in parasitic nematodes (Criscione *et al.* 2007; Gilabert and Wasmuth, 2013).

The two cryptic species, *Anisakis simplex* (s. s.) and *Anisakis pegreffii*, were first detected according to the BSC rule by multiple allozyme loci, which provided evidence for their reproductive isolation and the apparent absence of gene flow between them (Nascetti *et al*. 1986; Mattiucci *et al*. 2014). Later, they were depicted as distinct phylogenetic lineages (according to the PSC rule), as inferred from concatenated sequence analyses of both mitochondrial and nuclear DNA loci (Valentini *et al*. 2006; Mattiucci *et al*. 2014) and by the Procruster Rotation Analysis (PR), combining molecular/genetic datasets with morphological/morphometric traits of adult specimens of the two *Anisakis* species (Mattiucci *et al*. 2014). The two species were also found to differ in their geographical distribution (Nascetti *et al.* 1986; Mattiucci *et al.* 1997, 2014); however, their ranges overlap in some oceanic basins, such as the NE Atlantic Ocean (Spanish-Portuguese coast) and the Western Pacific Ocean (East China Sea and Sea of Japan) (Mattiucci *et al*. 1997, 2014; Abollo *et al.* 2001; Pontes *et al.* 2005; Mattiucci and Nascetti, 2008; Lee *et al*. 2009; Suzuki *et al.* 2010; Chou *et al.* 2011; Quiazon *et al*. 2011). In those areas, co-infection by *Anisakis* species pairs can occur in both pelagic and demersal fish hosts as well as in oceanic dolphin species (Mattiucci *et al.* 1997, 2004, 2008, 2014; Abollo *et al*. 2001; Pontes *et al.* 2005; Farjallah *et al.* 2008; Mattiucci and Nascetti, 2008). The occurrence of specimens, sometimes indicated as ‘putative hybrids’, or ‘recombinant genotypes’ or just ‘hybrids’, between the two species *A. simplex* (s. s.) and *A. pegreffii* has been reported by using polymerase chain reaction – restriction fragment length polymorphisms (PCR–RFLPs) of the internal transcribed spacer (ITS) region of ribosomal DNA (rDNA) and sequence analysis of the same gene. These specimens were either larval stages collected from various fish species or adults from cetaceans, from both allopatric (such as the Mediterranean Sea) (Farjallah *et al*. 2008; Meloni *et al*. 2011; Chaligiannis *et al.* 2012; Cavallero *et al.* 2012, 2014; Pekmezci *et al.* 2014) and sympatric areas (Abollo *et al*. 2003; Martìn-Sànchez *et al*. 2005; Umehara *et al*. 2006; Lee *et al*. 2009; Suzuki *et al.* 2010; Chou *et al.* 2011; Quiazon *et al*. 2011; Molina-Fernández *et al.* 2015).

Unfortunately, the above studies used a single nuclear marker (the ITS region of rDNA) and, consequently, lacked the power to decipher whether or not the observed shared polymorphism between the two taxa was caused by incomplete lineage sorting, historical introgression or current hybridization. As a result, whether *A. simplex* (s. s.) and *A. pegreffii* can hybridize remained an open question.

In the present paper, a Bayesian clustering of genotypes obtained from multi-marker nuclear loci analysis of individual nematodes of *Anisakis* spp. collected from allopatric and sympatric areas of the two species [*A. pegreffii* and *A. simplex* (s. s.)] was carried out in order to: (1) correctly identify specimens belonging to the parental taxa [*A. pegreffii and A. simplex* (s. s.)]; (2) compare the assignment of each *Anisakis* specimen obtained by biparentally inherited codominant nuclear markers (allozyme loci) and the sequence analysis of a new nuclear DNA locus (i.e. partial sequence of the elongation factor 1 alpha1 gene) with respect to the assignment obtained, on the same specimens, by means of the single molecular marker extensively used to distinguish the two species (i.e. the analysis of ITS region of rDNA); (3) recognize hybrid categories between the two taxa; and (4) distinguish introgression or current hybridization phenomena between the two species.

## MATERIALS AND METHODS

### Sampling

**Fig. 1.**
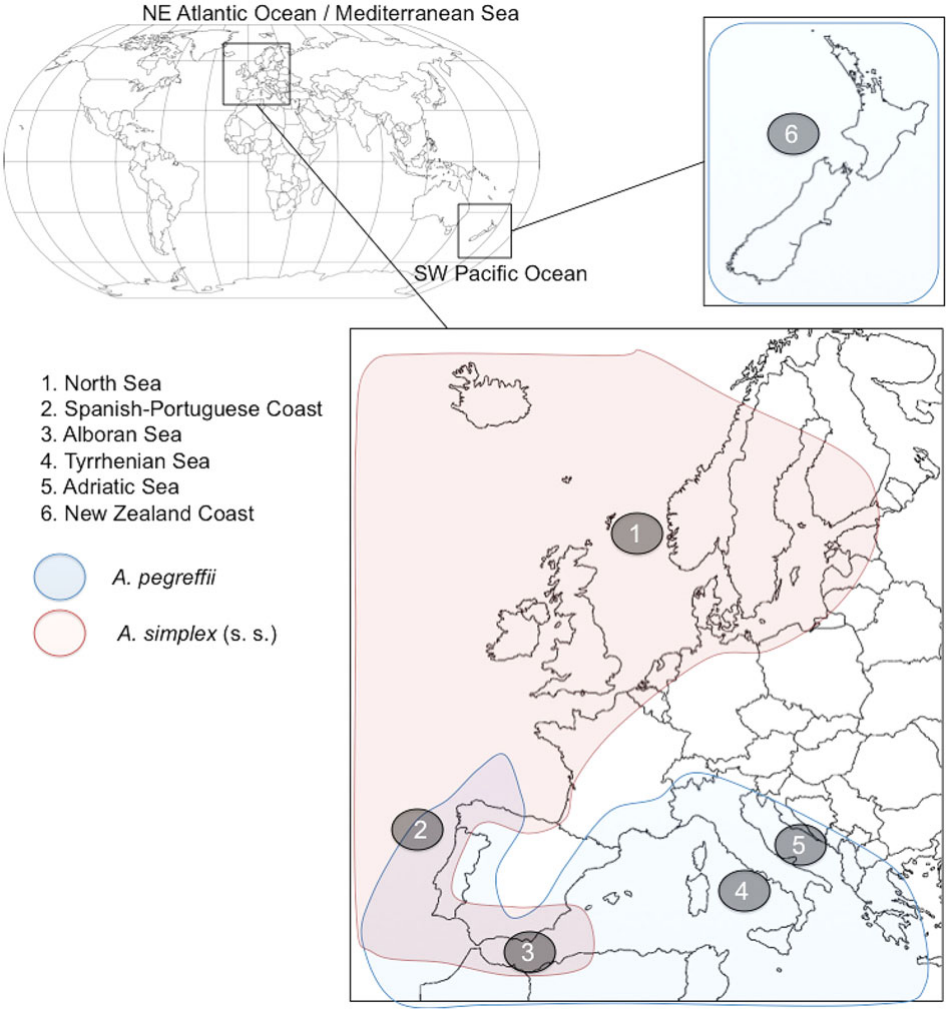
Map of the geographical areas from where the sampling of larvae and adults of *A. simplex* (s. s.) and *A. pegreffii* (as referred to in Table 1) was carried out.

A total of 689 specimens of *Anisaki*s spp. were examined. They were collected as the L3 larval stage from intermediate/paratenic fish hosts and as adults from cetacean species in sampling localities of the NE Atlantic Ocean, Mediterranean Sea and SW Pacific Ocean (Fig. 1, and Table 1). These localities are included in the geographical ranges of two species *A. pegreffii* and *A. simplex* (s. s.), from where they are reported both as allopatric [i.e. the North Sea for *A. simplex* (s. s.) and the South West Pacific Ocean for the species *A. pegreffii*] or as sympatric (i.e. the NE Atlantic Ocean off the coast of Portugal and Spain, Alboran Sea), according to Mattiucci *et al.* (1997, 2004, 2008, 2014), Abollo *et al.* (2001), Mattiucci and Nascetti (2008), Levsen and Lunestad (2010), Levsen and Karl (2014). Details concerning the sampling localities of the intermediate/paratenic (fish) and definitive hosts (cetaceans) of the two *Anisakis* species examined in this study are given in Table 1. Some of the nematodes were obtained from the frozen collection of anisakids stored in the Section of Parasitology, Department of Public Health and Infectious Diseases of ‘Sapienza University in Rome’, whereas new samples were collected during the years 2013–2014 from fish in the framework of the Project PARASITE and belong to the PARASITE-Biobank Node at the Section of Parasitology (Sapienza-University). In addition, the collection of adults was undertaken during the years 2011–2012 from stranded cetaceans, i.e. the pilot whale, *Globicephala melas* Traill, in the Southern Pacific Ocean (off the coast of New Zealand) and the striped dolphin, *Stenella coeruleoalba* (Meyen), stranded off the Italian coast. Nematodes collected from the stomach of their hosts were repeatedly washed in saline solution and preserved by freezing at −70° C in distilled water or stored in 70% alcohol.

**Table 1.** Sampling area, host species and life-history stage of the specimens studied from allopatric and sympatric areas of the nematodes *Anisakis pegreffii* and A. simplex (s. s.)

Sampling area	Host species	*N*	Life-history stage
NE Atlantic Ocean			
1. North Sea (59°13′N–00°14′W)	*Clupea harengus*	8	L3
	*Micromesistius poutassou*	28	L3
	*Scomber scombrus*	68	L3
2. Spanish-Portuguese Atlantic coast (41°48′N–9°44′W)	*Merluccius merluccius*	157	L3
Mediterranean Sea			
3. Alboran Sea (36°31′N–3°26′W)	*Lophius piscatorius*	9	L3
	*Scomber scombrus*	5	L3
	*Merluccius merluccius*	10	L3
4. Tyrrhenian Sea (41°7′N–13°24′E)	*Lepidopus caudatus*	23	L3
	*Merluccius merluccius*	29	L3
	*Scomber scombrus*	19	L3
5. Western Adriatic Sea (42°18′N–15°35′E)	*Stenella coeruleoalba*	137	A
	*Engraulis encrasicolus*	3	L3
	*Lophius piscatorius*	5	L3
	*Merluccius merluccius*	18	L3
	*Trachurus trachurus*	26	L3
	*Scomber scombrus*	35	L3
SW Pacific Ocean			
6. New Zealand coast (44°30′S–172°58′E)	*Globicephala melas*	109	A

A = adult; L3 = 3^rd^ stage larvae; *N* = number of parasites analysed by: allozymes, sequence analysis of the EF1 *α*−1 *n*DNA partial gene, and PCR–RFLPs of the ITS rDNA region.

### Laboratory procedures

Individual multi-locus genotypes of the 689 sampled individuals of *Anisakis* spp. were obtained by using 3 distinct classes of molecular markers: allozymes, sequence analysis of the elongation factor EF1 *α*-1 nuclear DNA gene and RFLP analysis of the ITS rDNA region (as well as sequences analysis of the same gene).

Standard horizontal starch gel electrophoresis was performed to analyse variation at 4 allozyme loci of diagnostic value for the studied species (Mattiucci *et al.* 1997, 2014). These loci were: adenylate kinase (*Adk-2*, EC 2·7·4·3), leucine–alanine peptidase (*Pep C-1, Pep C-2*, EC 3·4·11) and superoxide dismutase (*Sod-1*, EC 1·15·1·1). The procedures of starch gel electrophoresis used in this study were described in detail by Mattiucci *et al*. (1997).

Tissue homogenates, obtained during the starch gel electrophoresis procedures, were preserved at −20 °C and subsequently used to obtain genomic DNA extracts of each examined individual. The total DNA was extracted using the cetyltrithylammonium bromide method, as detailed elsewhere (Mattiucci *et al*. 2014). DNA obtained was quantified by using the Qubit^™^ dsDNA HS Assay Kit with Qubit 2·0 (Invitrogen^™^).

For sequencing the ITS rDNA region, PCR amplification was performed using the primers NC5 (5′-GTAGGTGAACCTGCGGAAGGATCATT-3′) and NC2 (5′-TTAGTTTCTTTTCCTCCGCT-3′) according to the procedure reported in Zhu *et al.* (2000) and Abollo *et al*. (2003). PCR amplification conditions were the following: 95 °C for 10 min (initial denaturation), followed by 30 cycles at 95 °C for 30 s (denaturation), 55 °C for 30 s (annealing), 72 °C for 75 s (extension) and a final elongation step at 72 °C for 7 min. Three *μ*L of the amplification products were visualised on 1% Gel-Red (Biotium) stained agarose gels to check the quality of amplification. PCR products were then digested using the *Hinf*I restriction endonuclease according to the standard procedures (D'Amelio *et al.* 2000; Abollo *et al.* 2003; Pontes *et al.* 2005). The digestion was performed using 1 *µ*L of restriction enzymes (*Hinf*I), 1 *µ*L of BSA (Bovine Serum Albumin, Acetylated, 10 mg mL^−1^), 1 *µ*L of buffer B 10× and 7 *µ*L of PCR products up to a final volume of 10 *µ*L. The digestion was performed for 3 h at 37 °C. Restriction patterns were visualized on 3% agarose gels under UV-light. Further confirmation of the heterozygote restriction patterns produced by digestion with *Hinf*I for some individuals was obtained by sequencing the ITS rDNA using those PCR primers.

For the elongation factor (EF1 *α*−1 nDNA) nuclear gene, oligonucleotide primers were designed based on the elongation factor 1 alpha1 gene of genomic DNA sequence deposited in the GenBank database under the accession number KP326558. The primers used in the present paper were designed by hand and verified by means of the on-line software program Primer3 (http://bioinfo.ut.ee/primer3-0.4.0/). Thus, PCR amplification was performed using the primers EF-F (5′-TCCTCAAGCGTTGTTATCTGTT-3′) and EF-R (5′-AGTTTTGCCACTAGCGGTTCC-3′). PCRs were carried out in a 25 *µ*L volume containing 0·5 *µ*L of each primer 10 mm, 2·5 *µ*L of MgCl_2_ 25 mm (Promega), 1·5 *µ*L of 5× buffer (Promega), DMSO 0·08 mm, 0·5 *µ*L of dNTPs 10 mm (Promega), 5 U of Go-*Taq* Polymerase (Promega) and 2 *µ*L of total DNA. PCR temperature conditions were the following: 94 °C for 3 min (initial denaturation), followed by 35 cycles at 94 °C for 45 s (denaturation), 58 °C for 40 s (annealing), 72 °C for 1 min (extension) and followed by post-amplification at 72 °C for 10 min. An initial sample of 50 individuals, belonging to the two species, *A. pegreffii* and *A. simplex* (s. s.), previously identified by allozymes, were sequenced at the elongation factor 1 alpha1 gene. The sequences obtained were aligned in order to detect fixed diagnostic nucleotide positions able to discriminate the two species under study. Then, all the specimens analysed in the present paper were sequenced at the EF1 *α*−1 nDNA gene.

### Data analysis

The sequences obtained from ITS rDNA and EF1 *α*−1 nDNA region were aligned by using Clustal X version 2.0 software (Larkin *et al.* 2007). ITS rDNA sequences were also analysed by Genbank Blast software, in order to verify their similarity with respect to the species under study, and aligned with those from the same species previously obtained (Mattiucci *et al*. 2014) by using Clustal X 2·0 (Larkin *et al*. 2007).

All subsequent population genetic analyses were carried out on individual multi-locus genotypes based on the four allozyme loci, and the pattern of variation at diagnostic nucleotide positions of the EF1 *α*−1 *n*DNA region (i.e. 5 distinct loci overall). The occurrences of the expected Hardy–Weinberg equilibrium for each locus and the genotypic linkage equilibrium between each pair of loci were assessed by means of exact tests, as implemented in the software GENEPOP (web version; Rousset, 2008). Significance levels were adjusted using the sequential Bonferroni correction for multiple tests (Rice, 1989). To investigate the population genetic structure in our dataset inferred from those five distinct loci (allozymes and EF1 *α*−1 nDNA region) and to identify instances of gene exchange between species, a Bayesian clustering algorithm was used by means of the program STRUCTURE 2·3·3 (Pritchard *et al.* 2000). STRUCTURE is a model-based procedure that uses individual multi-locus genotypes to identify the optimal number of clusters (*K*) in a dataset, by minimizing the resulting Hardy–Weinberg and linkage disequilibria. The analysis was run setting the predefined number of clusters between 1 and 6 (i.e. the number of sampling areas in our dataset). Ten replicates of the analysis were carried out to check for consistency, each run for 100 000 MCMC iterations, following a burn-in of 50 000 iterations, under the admixture model and the assumption of correlated allele frequencies among populations. The best *K* value was identified using both the log probability of the data and the rate of change in the log probability of the data between successive *K* values as optimality criteria (Evanno *et al.* 2005).

The results obtained with the Bayesian clustering algorithm on those loci were compared with the results inferred from the single-locus approach based on the conventional PCR–RFLPs analysis of the single ITS rDNA marker in order to check for consistency. However, the genotypes inferred from PCR–RFLPs analysis of ITS rDNA were not included in the analysis of STRUCTURE, because the ITS region of rDNA undergoes concerted evolution (Elder and Turner, 1995; Ganley and Kobayashi, 2007), a phenomenon which violates the assumption of Hardy–Weinberg, necessary to perform elaboration by STRUCTURE.

## RESULTS

### Allozyme markers

**Fig. 2.**
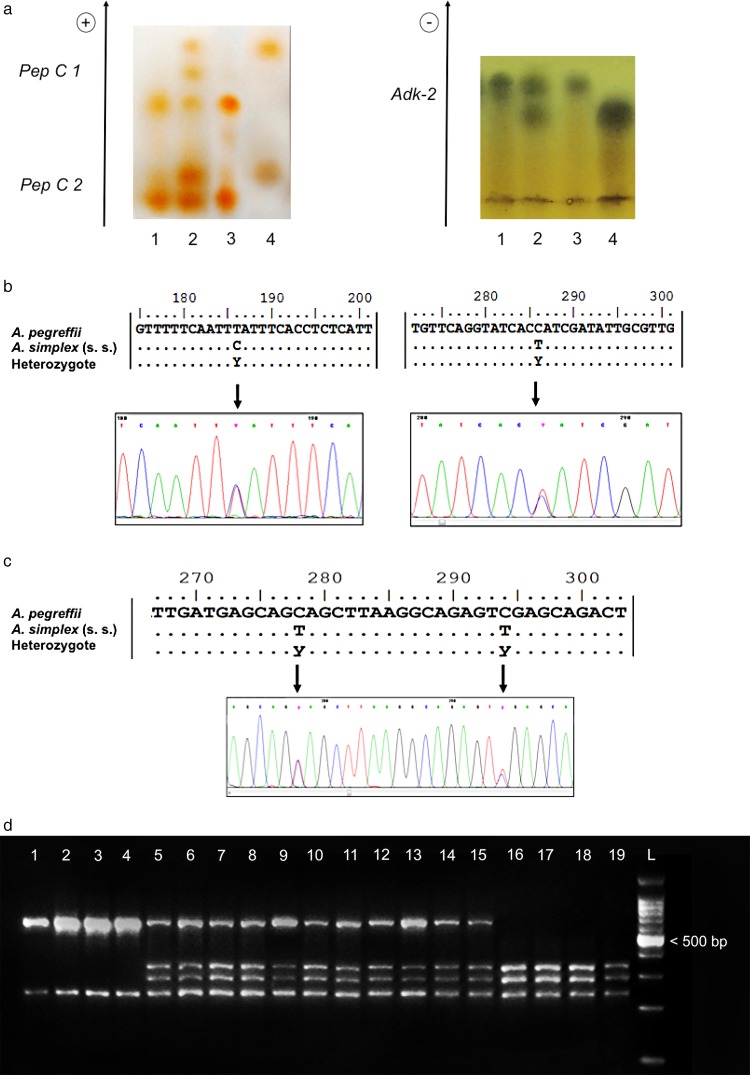
Genotypes at each single nuclear genetic marker used in the present study. (a) Allozyme patterns obtained using enzymes *Pep C-1, Pep C-2* and *Adk-2*; plate 1: *Pep C-1* (dimeric structure, two alleles); specimen nos. 1 and 3: *A. simplex* (s. s.) homozygote pattern *90*/*90*; specimen no. 4: *A. pegreffii* homozygote pattern *100*/*100*; specimen no. 2: heterozygote pattern *90*/*100; Pep C-2* (monomeric structure, two alleles); specimen nos. 1 and 3: *A. simplex* (s. s.) homozygote pattern *96/96*; specimen no. 4: *A. pegreffii* pattern *100*/*100*; specimen no. 2: heterozygote pattern *96*/*100*; plate 2: *Adk-2* (monomeric structure, two alleles); specimen nos. 1 and 3: *A. simplex* (s. s.) homozygote pattern *105*/*105*; specimen no. 4: *A. pegreffii* homozygote pattern *100*/*100*; specimen no. 2: heterozygote pattern *100/105*. (b) Alignment of the elongation factor (EF1 *α*−1 *n*DNA) nuclear gene in the two species *A. pegreffii* and *A. simplex* (s. s.). The arrows show the fixed diagnostic nucleotide positions detected between the two species. A heterozygote pattern at both positions is shown. The relative electropherogram of this genotype is also reported below. Dots indicate identity; standard IUPAC codes were used, i.e. Y = C/T;(c) PCR–RFLP patterns obtained by digestion of the ITS region of rDNA with the restriction enzyme *Hinf*I (according to D'Amelio *et al.* 2000) showing: specimens nos. 1–4: *A. simplex* (s. s.) genotype (two bands); specimen nos. 16–19: *A. pegreffii* genotype (three bands); specimen nos. 5–15 heterozygote genotypes (four bands); L: 100 bp ladder.(d) Alignment of the ITS-1 region of rDNA and electropherogram of the same gene indicated by arrows, representing the two diagnostic positions (according to D'Amelio *et al.* 2000; Abollo *et al*. 2003) between the two species. The electropherogram of the heterozygote genotype is also shown below. Dots indicate identity; standard IUPAC codes are used, i.e. Y = C/T.

According to the alleles observed at the diagnostic loci, i.e. *Adk-2^100^, Pep C-1^100^, Pep C-2^100^* (Fig. 2a) and *Sod-1^100^,* 487 specimens were assigned to the species *A. pegreffii*; whereas, according to the diagnostic alleles *Adk-2^105^, Pep C-1^90^, Pep C-2*^96^ and *Sod-1^105^* (Fig. 2a), as indicated in Mattiucci *et al.* (2014), 191 larvae corresponded to the species *A. simplex* (s. s.) (Table 2). Eleven larval specimens, collected in fish from the Spanish Atlantic coast (*N* = 10) and in a sample from the Alboran Sea (*N* = 1), showed a heterozygote genotype between *A. pegreffii* and *A. simplex* (s. s.) (Fig. 2a) at all the diagnostic allozyme loci (i.e. *Adk-2^100/105^, Pep C-1^90/100^, Pep C-2^96/100^* and *Sod-1^100/105^*) (Table 2, Fig. 2a).

**Table 2.** Assignment to the parental taxa, i.e. *Anisakis pegreffii* and Anisakis simplex (s. s.), or mixed ancestry, of nematode specimens, according to their genotypes observed at each single nuclear marker used in the present study

				Allozymes									EF1 *α*−1 (*n*DNA)			PCR–RFLPs ITS rDNA		
	*Adk-2*			*Pep C-1*			*Pep C-2*			*Sod*								
	AS	AP	He	AS	AP	He	AS	AP	He	AS	AP	He	AS	AP	He	AS	AP	He
1. North Sea	104	–	–	104	–	–	104	–	–	104	–	–	104	–	–	103	–	1
2. Spanish-Portuguese Atlantic coast	87	60	10	87	60	10	87	60	10	87	60	10	87	60	10	87	57	13
3. Alboran Sea	–	23	1	–	23	1	–	23	1	–	23	1	–	23	1	–	23	1
4. Tyrrhenian Sea	–	71	–	–	71	–	–	71	–	–	71	–	–	71	–	–	67	4
5. Western Adriatic Sea	–	224	–	–	224	–	–	224	–	–	224	–	–	224	–	–	215	9
6. New Zealand coast	–	109	–	–	109	–	–	109	–	–	109	–	–	109	–	–	107	2

AS, genotype of the parental species Anisakis simplex (s. s.); AP, genotype of the parental species *Anisakis pegreffii*; He, heterozygote genotype of a mixed ancestry between the two Anisakis species.

### Elongation factor 1 alpha1 nDNA subunit partial gene region

A fragment of 409 bp in length of the EF1 *α*−1 nDNA region was obtained for all of the 689 specimens analysed. It revealed the presence of two diagnostic nucleotide sites between *A. pegreffii* and *A. simplex* (s. s.) (Fig. 2b). These positions were: 186, showing a T in *A. pegreffii*, whereas it was C in the parental taxon *A. simplex* (s. s.); and the position 286, showed a C in *A. pegreffii* but always a T in *A. simplex* (s. s.) (Fig. 2b).

According to the diagnostic positions detected, 487 specimens were assigned to the species *A. pegreffii,* whereas 191 were assigned to *A. simplex* (s. s.) (Table 2). In addition, 11 specimens showed a heterozygote genotype (C/T) at both diagnostic positions, i.e. 186 and 286, showing two overlapping C/T peaks in the sequences analysed (Fig. 2b).

Sequences of the EF1 *α*−1 nDNA region were deposited in GenBank under the accession numbers KT825684 for *A. pegreffii,* and KT825685 for *A. simplex* (s. s.).

### PCR–RFLPs of ITS region of rDNA

The entire ITS1–5·8S-ITS2 rDNA region was amplified and a fragment of 905 bp obtained from the same 689 *Anisakis* spp. individuals analysed at both diagnostic allozymes and the EF1 *α*−1 *n*DNA genes. Restriction with *Hinf*I produced bands at 370, 300 and 250 bp, corresponding to *A. pegreffii* in 469 specimens (Table 2, Fig. 2c), whereas fragments of 250 and 620 bp in 190 specimens were found to belong to *A. simplex* (s. s.) (Table 2, Fig. 2c). Thirty *Anisakis* specimens exhibited a different restriction pattern using PCR–RFLP analysis. Here, the restriction with *Hinf*I digestion produced fragments of 620, 370, 300 and 250 bp that were heterozygote genotypes between *A. pegreffii* and *A. simplex* (s. s.) (Fig. 2c). The results achieved using the PCR–RFLPs of rDNA were confirmed by the aligned sequences at the ITS region of all the 689 specimens analysed (Fig. 2c). The sequences obtained matched the sequences previously deposited in GenBank for *A. pegreffii* or *A. simplex* (s. s.). Thus, following that putative diagnostic marker, 190 individuals (in fish hosts from the North Sea and off the Spanish-Portuguese coast) matched the sequence corresponding to the species *A. simplex* (s. s.), whereas 469 specimens corresponded to *A. pegreffii.*

Finally, 30 individuals, characterized by the presence of two overlapping C/T peaks in those diagnostic nucleotide positions (i.e. 278 and 294 of the ITS1 region of rDNA), confirmed a pattern of heterozygote genotypes (Fig. 2d). They included *Anisakis* specimens from two sympatric areas of the two species (i.e. the Spanish Atlantic coast and the Alboran Sea) (Fig. 1, Table 2). Thirteen heterozygote individuals were also recognized in the samples collected from the Mediterranean Sea, i.e. in areas nos. 4 and 5; and also in specimens collected in extreme allopatric areas (sample no. 1, North Sea; and no. 6, New Zealand) (Table 2 and Fig. 1).

However, among those 30 individuals showing the heterozygote pattern using the PCR–RFLPs of rDNA (Fig. 2d), only 11 individuals presented a heterozygote genotype at the other nuclear diagnostic loci (i.e. *Adk-2, Pep C-1, Pep C-2, Sod* and EF1 *α*−1 nDNA region) (Table 2).

### Multi-marker genotyping data analysis by STRUCTURE

Considering the discordance in the species assignments [i.e. to *A. pegreffii* or *A. simplex* (s. s.)] or heterozygote patterns obtained by the five nuclear loci (*Adk-2, Pep C-1, Pep C-2, Sod-1* and EF1 *α*−1 *n*DNA) studied with respect to those obtained by PCR–RFLPs of rDNA of the ITS (Table 2), all the genotypes obtained at those five loci (*Adk-2, Pep C-1, Pep C-2, Sod-1* and EF1 *α*−1 nDNA) were tested for the occurrence of the expected Hardy–Weinberg equilibrium for each locus and the genotypic linkage equilibrium between each pair of loci. Strongly significant deviations from the expected Hardy–Weinberg equilibrium (*P* < 0·0001) were observed for all the studied loci within the sample from area no. 2 (off the Spanish-Portuguese Atlantic coast), whereas none of the other samples showed deviations. Similarly, the sample collected from off the Spanish-Portuguese Atlantic coast (no. 2) was the only one where significant linkage disequilibria were found among all pairs of loci (all *P* < 0·0001); in contrast, no statistically significant disequilibrium was observed within the other *Anisakis* samples.

**Fig. 3.**
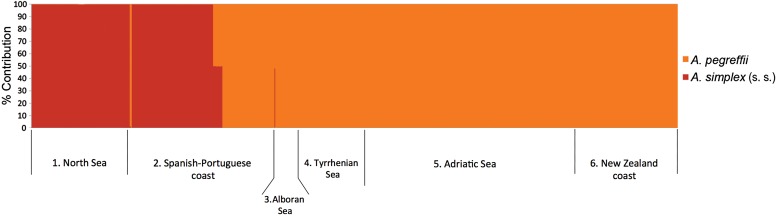
Percentage contribution (Q value) of *Anisakis pegreffii* and *A. simp*lex (s. s.) to the multi-locus genotype of each studied individual (barplots) estimated using STRUCTURE (a) with *k* = 2. The numbers refer to the sampling areas (see Fig. 1 and Table 1), as follows: 1: North Sea; 2: Spanish-Portuguese Atlantic coast; 3: Alboran Sea; 4: Tyrrhenian Sea; 5: Adriatic Sea; 6: New Zealand coast.

The population genetic structure in our dataset and instances of gene exchange between *A. pegreffii* and *A. simplex* (s. s.) were then assessed on the basis of a Bayesian clustering algorithm implemented in the program STRUCTURE 2·3·3 (Pritchard *et al.* 2000). Using both the highest ln-probability and the delta-*K* (Evanno *et al.* 2005) optimality criteria, the STRUCTURE analysis indicated *K* = 2 as the clustering option which best fitted the data (see Supplementary Fig. 1). Considering the five diagnostic nuclear loci here studied (*Adk-2, Pep C-1, Pep C-2, Sod-1* and EF1 *α*−1 nDNA partial region), with that clustering option, all the individuals from samples no. 4 (Tyrrhenian Sea), no. 5 (Western Adriatic Sea), no. 6 (New Zealand coast) and all except one larval specimen from sample no. 3 (Alboran Sea) were assigned with high confidence level (>90%) to *A. pegreffii* (Fig. 3). All individuals from sampling locality no. 1 (North Sea) were assigned to *A. simplex* (s. s.). Nevertheless, among the 157 *Anisakis* spp. individuals from sample no. 2 (Spanish-Portuguese Atlantic coast), 87 (56%) were identified as belonging to *A. simplex* (s. s.), 60 (38%) were assigned to *A. pegreffii* and 10 (6%) were identified as individuals of mixed ancestry, having, in all cases, a *Q*-value = 0·50 (Fig. 3). A mixed ancestry between the two taxa was also inferred for one other specimen from the Alboran Sea (sample no. 3) (Fig. 3). The 11 individuals identified as admixed by STRUCTURE were those showing a clear heterozygote patterns at all the diagnostic loci (Table 2), corresponded to the F_1_ hybrid class. However, the same 11 F_1_ individuals showed also the heterozygote pattern when using the PCR–RFLPs of rDNA (Tables 2 and 3; Fig 2c).

**Table 3.** Identification of specimens belonging to the parental taxa, i.e. *Anisakis pegreffii* and A. simplex (s. s.), or their F_1_ hybrids, as inferred by STRUCTURE analysis, based on genotypes obtained from allozymes diagnostic loci and E*α*−1 nDNA

Sampling area	Host species	*A. pegreffii*	*A. simplex (*s. s.)	F_1_ hybrid
NE Atlantic Ocean				
1. North Sea	*Clupea harengus*	–	8	–
	*Micromesistius poutassou*	–	28	–
	*Scomber scombrus*	–	68	–
2. Spanish-Portuguese Atlantic coast	*Merluccius merluccius*	60	87	10
Mediterranean Sea				
3. Alboran Sea	*Lophius piscatorius*	9	–	–
	*Scomber scombrus*	5	–	–
	*Merluccius merluccius*	9	–	1
4. Tyrrhenian Sea	*Lepidopus caudatus*	23	–	–
	*Merluccius merluccius*	29	–	–
	*Scomber scombrus*	19	–	–
5. Western Adriatic Sea	*Stenella coeruleoalba*	137	–	–
	*Engraulis encrasicolus*	3	–	–
	*Lophius piscatorius*	5	–	–
	*Merluccius merluccius*	18	–	–
	*Trachurus trachurus*	26	–	–
	*Scomber scombrus*	35	–	–

SW Pacific Ocean				
6. New Zealand coast	*Globicephala melas*	109	–	–

## DISCUSSION

The genotyping approach based on multiple-nuclear loci not only provides a powerful means to clarifying taxonomic issues in parasitic taxa, determining their population structure, estimating gene flow and demonstrating reproductive isolation, but it also distinguishes current hybridization and introgressive hybridization phenomena. Among the nuclear codominant markers, allozymes provided valuable genetic data for distinctiveness, reproductive isolation and absence of gene flow in sympatric cryptic species of anisakid nematodes (Mattiucci and Nascetti, 2008). When sister taxa start to diverge from a common ancestor, allele frequencies at each allozyme locus are initially similar, but, over time, the genetic drift gradually results in divergence of those frequencies and, eventually, also in the appearance of new alleles in each population through mutation. Consequently, separate populations can acquire different alleles at some loci. This could be the case for those fixed allele differences found between the two *Anisakis* taxa at those allozyme loci (Mattiucci *et al*. 2014).

Similarly, at the level of sequence variation in nuclear loci, such as the EF1 *α*11 nDNA gene region, it is demonstrated here that the two taxa *A. pegreffii* and *A. simplex* (s. s.) exhibited species-specific fixed nucleotide variation at some positions. Those fixed difference in the EF1 *α*−1 nDNA partial gene were found in a large number of specimens belonging to the two parental taxa [i.e. *A. pegreffii* and *A. simplex* (s. s.)] collected from both allopatry and sympatry. The large sample sizes of parental populations belonging of the two species confirm the fixation of those diagnostic positions at the partial region of the EF1 *α*−1 *n*DNA gene. As a consequence, having validated this marker in a large number of specimens, it represents a novel nuclear marker of diagnostic value for the recognition of the two cryptic species.

Assuming that a common approach to identify hybrids is appropriate, the use of species-specific markers (fixed with alternative alleles in the parental ‘taxa’) requires an adequate sample size of the ‘pure’ parental taxa investigated; this prerequisite was fulfilled in the present study, with a large number of individuals tested from allopatric and sympatric areas for all of the fixed diagnostic loci. Secondly, one could say that geographical differences in the allele frequencies found in the parental taxa could confound the fixation of those alleles considered as alternatives of diagnostic value between the ‘pure’ taxa, if those ‘pure parental’ populations are geographically separated from the hybrid populations. The latter is not the case for the two *Anisakis* spp. under study, because those fixed differences at those nuclear loci (allozymes and EF1 *α*−1 nDNA) were validated in both allopatric and sympatric populations, both in the present study and in previous ones (Mattiucci *et al.* 1997, 2014). In addition, the F_1_ hybrids found were observed in syntopy in both parental species in, at least, their intermediate fish hosts.

Indeed, the Bayesian clustering analysis, using nuclear allozyme loci (*Adk-2, Pep C-1, Pep C-2* and *Sod-1*) and the EF1 *α*−1 nDNA partial region, allowed us to either correctly define ‘pure’ individuals belonging to the two parental taxa, i.e. *A. simplex* (s. s.) or *A. pegreffii*, or to unravel patterns of natural hybridization between the two species. According to this, 10 specimens collected from the fish host in the geographical area where the two species overlap (i.e. the sympatric region of the Spanish-Portuguese Atlantic coast), and 1 individual from the Alboran Sea (considered as the Atlantic basin rather than the Mediterranean one), were found to belong to the F_1_ hybrid category. These resulted from a current hybridization event between sympatric *A. simplex* (s. s.) and *A. pegreffii*. These hybrid nematodes were indeed heterozygous for all of the diagnostic allozyme loci and for the diagnostic nucleotide positions observed in the EF1 *α*–1 *n*DNA gene (Fig. 2). All of the F_1_ hybrids identified were at the larval stage; no F_1_ hybrid at the adult stage was found in the present study.

Our findings provide new insights on the hybridization of *A. pegreffii* and *A. simplex* (s. s.), which results in a contemporary interbreeding phenomenon between sympatric specimens of the two species. The frequency of current hybridization, so far observed, appears to be about 5·5%. In those sympatric geographical areas, it is known that the two parasite species can be often found in mixed infections in the same individual cetacean host (Mattiucci *et al*. 2014). Furthermore, considering that the number of mature females of these anisakids is generally higher than that of mature males in their definitive hosts (Mattiucci, personal communication), it could be that when the intensity of the infection by one of these two species is low, mating events between the two species could occur, resulting in a first generation (F_1_) of larval hybrids. In the case of the F_1_ hybrids identified here, 10 resulted from the mating of female *A. simplex* (s. s.) with male of *A. pegreffii*, causing a maternal inheritance of the mtDNA of *A. simplex* (s. s.), and one exhibited a *A. pegreffii* matrilineage (data obtained from the sequence analysis of the mtDNA *cox2* region; data not shown). On the other hand, it has been suggested that there is a tendency for hybridization to take place preferentially between parental species differing greatly in abundance. This hypothesis suggests that the absence of conspecific pairing partners and mating stimuli for females of rarer species may be important factors in increasing the likelihood of interspecific current hybridization (Avise and Saunders, 1984).

Larval stages of F_1_ hybrids between *A. pegreffii* and *A. simplex* (s. s.) have previously been recorded, respectively, from the horse mackerel, *Trachurus trachurus* (see Mattiucci *et al*. 2008) and European hake, *Merluccius merluccius* (see Mattiucci *et al.* 2004; Cipriani *et al.* 2015) collected in a sympatric region of Spanish Atlantic waters. However, even if these larvae reach the definitive host, the lack of other hybrid generations (backcrosses), as seen in the present study, appears to support the hypothesis that some selective factors decrease F_1_ hybrid fitness. Laboratory crosses might contribute to clarifying these aspects of a natural hybridization between the two taxa, as performed for other parasitic helminths (Théron *et al.* 1989). However, while the *in vitro* culture of the two species of *Anisakis*, from the L3 stage to their adult stage, is clearly possible (Nascetti *et al*. 1986), other steps in the lifecycle of these parasites, including the infection of the first intermediate hosts, have never been carried out.

Contemporary hybridization events (i.e. F_1_ generation) appear to be a current phenomenon happening between other cryptic species of anisakid nematodes. Indeed, F_1_ adult nematodes were detected by multi-loci allozyme analysis between *Pseudoterranova decipiens* (s. s.) and *P. krabbei*, often occurring in sympatry and syntopy in the same pinniped as definitive hosts from NE Atlantic waters (Paggi *et al.* 1991). Similarly, the occurrence of F_1_ hybrids was observed between the two cryptic species, *Contracaecum rudolphii* sp. A and *C. rudolphii* sp. B from fish-eating birds (cormorant, *Phalacrocorax carbo sinensis*) in areas of sympatry (Mattiucci and Nascetti, unpublished results). Other cryptic species of nematodes, such as *Paramacrostrongylus* spp. from species of kangaroo, are able to hybridize in an area where the ranges of the two host species overlap (Chilton *et al.* 1997). Even in the case of highly separated taxa, such as between the two cryptic ascaridoid species of equids, *Parascaris univalens* and *Parascaris equorum,* there is some evidence that they might hybridize (Biocca *et al.* 1978; Bullini *et al.* 1978); however, they produce infertile offspring (Goday and Pimpinelli, 1986). To our knowledge, other examples of contemporary hybridization have been detected between *Ascaris lumbricoides* and *Ascaris suum*; indeed, Bayesian clustering based on microsatellite data revealed evidence for hybridization in sympatric populations from Guatemala and China (Criscione *et al.* 2007; Detwiler and Criscione, 2010). Similarly, in other helminth species, such as *Schistosoma mansoni* and *S. rodhaini*, the use of Bayesian clustering methods based on microsatellite datasets has demonstrated the occurrence of recent cross-transmission events between the two taxa (Steinauer *et al*. 2010).

In terms of ecological processes associated with parasite hybridization, different possible host–hybrid–parasite interactions can result (Fritz *et al.* 1999). Evidence of current hybridization between parasite taxa raises questions with regard to the epidemiology and ecological aspects of these parasites, such as a differential potential transmission of hybrids to a new host, a greater or lower adaptation to an intermediate host, or a potentially wider geographical range of the ‘parental’ taxa resulting from a greater adaptation to both biotic and abiotic factors. It has indeed been observed that some hybrids can colonize new host species, or exhibit an increased level of host infectivity and pathogenicity, exceeding those of either of the parental taxa. Furthermore, hybrid parasites have also exhibited different host susceptibility/resistance traits with respect to the parental taxa, and hybrid phenotypes of parasites have shown some changes in the epidemiological parameters, such as prevalence or density, within a geographical area or within definitive and intermediate hosts (Théron *et al.* 1989; Criscione *et al.* 2007; Volf *et al.* 2007; Dybdahl *et al.* 2008; Steinauer *et al.* 2008; Detwiler and Criscione, 2010).

The finding here of F_1_ hybrids between *A. simplex* (s. s.) and *A. pegreffii* appears to be not associated with a particular epidemiological pathway. All of the F_1_ larvae identified were collected from nine specimens of a demersal fish, the European hake *Merluccius merluccius,* caught in waters where the parasites occur in sympatry. The larvae were all found in co-infections with the two parental species, *A. pegreffii* and *A. simplex* (s. s.), except in one case. Most of the F_1_ (*N* = 9) larvae were found in the visceral body cavity of the host; whereas, only in two cases they were located in the flesh of the fish. A significant difference in the site of infection has been reported for these two species in *M. merluccius* for sympatric and syntopic populations, with *A. simplex* (s. s.) having a greater capacity to invade the musculature the fish (Cipriani *et al.* 2015). Further research, using a larger number of correctly identified hybrid individuals, might highlight different scenarios in some phenotypic traits, such as, for instance, a differential capacity of invading host tissues, between the F1 hybrids and the parental species. In other helminths, such as a species of the digenean genus *Microphallus*, the infectivity of F_1_ hybrid parasites was lower with respect to that exhibited by the parental species (Dybdahl *et al*. 2008); these authors suggested that the lower fitness of the hybrids was due to outbreeding depression in the hybrids.

The present study also demonstrates that current hybridization outside the sympatric areas of the two cryptic species does not occur. Indeed, in the eastern (Adriatic Sea) and western (Tyrrhenian Sea) Mediterranean, as well as in strict allopatric areas (New Zealand coast), no F_1_ hybrids were detected by the multi-loci approach. Indeed, the Bayesian clustering, based on allozymes and the EF1 *α*−1 nDNA gene, clearly indicated that all the specimens of *Anisakis* spp. from the Mediterranean Sea, except for one individual collected from the Alboran Sea, an area of sympatry of the two *Anisakis* species (Mattiucci *et al*. 2015). On the other hand, Alboran Sea is actually considered an Atlantic basin water rather than a Mediterranean one (Tintore *et al*. 1988). Similarly, those specimens collected in hosts from off the New Zealand coast (allopatric area), were assigned at a high probability level (100%) to the species *A. pegreffii*. Similarly, all the specimens collected from the North Sea were assigned, at a high probability level (100%), to the parental species *A. simplex* (s. s.). Conversely, if based only on the PCR-RFLP analysis of ITS rDNA, a certain number of ‘hybrids’ were identified in the present study from these allopatric areas mentioned above (Table 2). They were recognized at both larval and adult stages, not only in hosts from Spanish Atlantic coasts (*N* = 13) but also from various areas of the Mediterranean Sea (*N* = 16), and even in samples collected in strictly allopatric areas, i.e. New Zealand waters (*N* = 2) and the North Sea (*N* = 1). Nevertheless, Bayesian clustering demonstrated that, among those specimens, 19 were correctly assigned to one of the parental species, i.e. *A. pegreffii* or *A. simplex* (s. s.), when based on allozyme loci and the EF1 *α*−1 nDNA gene (Table 2). On the other hand, the finding of numerous larval and adult hybrid specimens has previously been reported by several authors in those areas of the Mediterranean Sea (Farjallah *et al.* 2008; Meloni *et al.* 2011; Cavallero *et al.* 2012, 2014; Chaligiannis *et al.* 2012; Pekmezci *et al.* 2014; Molina-Fernández *et al.* 2015), as just inferred from analyses of the ITS region of rDNA. In the present study, if the analysis of the ITS region of the rDNA gene had been the only conventional marker used in the identification of the parasites which we identified, a misidentification of these specimens of *Anisakis* would have resulted. It is likely that the two nucleotide positions found in the ITS-1 of rDNA ]i.e. 278 showing C in *A. pegreffii* and T in *A. simplex* (s. s.), and 294 showing C in *A. pegreffii* and T in *A. simplex* (s. s.)] are, according to D'Amelio *et al*. (2000), not actually fixed diagnostic markers. As a consequence, the nucleotide position 294, detected at the PCR–RFLPs analysis by *Hinf*I in the ITS1 rDNA, cannot be retained as a 100% diagnostic marker between the two species, as previously stated (D'Amelio *et al.* 2000), just because it is a shared polymorphism between the two taxa. This could be the likely the outcome of the incomplete lineage sorting of a shared ancestral variation, or the result of a historical introgression at this lone marker, resulting in a polymorphism in both *A. pegreffii* and *A. simplex* (s. s.), occurring at the ITS-1 region of rDNA.

### Concluding remarks

In retrospect, the present study has demonstrated that single molecular markers on their own based on the ITS region of rDNA were not always able to recognize ‘pure’ specimens belonging to the two cryptic taxa and, even less, successfully, their hybrid categories. On the other hand, as previously suggested for other helminth species (Criscione *et al*. 2007) the ITS (rDNA complex in general) is a multi-copy gene which undergoes concerted evolution phenomena; as a consequence, it lacks the power to disentangle hybridization events between closely related taxa (Ganley and Kobayashi, 2007).

However, based on the present results, the use of other nuclear markers (i.e. allozymes and the novel markers obtained from the EF1 *α*−1 nDNA gene) not only permitted the clear distinction of specimens belonging to the two cryptic species *A. simplex* (s. s.) and *A. pegreffii*, but also enabled the distinguishing of current hybridization and introgressive hybridization events. Indeed, data obtained from these nuclear markers suggest that no introgressive hybridization takes places between the two species; this conclusion is supported by evidence that no backcrossing with the two parental species – which represents subsequent generations of hybrids – were found in our study among the large number of sympatric and allopatric specimens examined. This finding suggests that, even if current hybridization might likely occur between *A. pegreffii* and *A. simplex* (s. s.) in some sympatric areas (i.e. the NE Atlantic waters off the Spanish-Portuguese coast and the Alboran Sea), the resulting offspring appear to have a reduced fitness. It is likely that either the F_1_ offspring is infertile, even if it reaches the adult stage, or that selection disadvantages the survival of hybrid offspring, or that some reproductive isolating mechanisms do not permit backcrossing of F_1_ hybrids with the parental types. As a consequence, the two species maintain their identity.

On the other hand, identification of these hybridization phenomena is essential to improving our understanding of the micro-evolutionary processes which have accompanied the speciation of these parasites. It is also important in terms of elucidating anisakid ecology, including the distributional ranges and overlap of the species. It will also shed light on the life-cycles of these parasites, including any intermediate/paratenic host preferences, epidemiological parameters of infection and their pathogenicity/virulence in natural and accidental hosts, such as humans. The latter is of particular importance, since the zoonotic disease anisakiasis has been so far reported as being caused by both *A. simplex* (s. s.) and *A. pegreffii* (D'Amelio *et al.* 1999; Umehara *et al*. 2007; Mattiucci *et al*. 2011, 2013).

Finally, the present study takes the advantage of the use of a Bayesian clustering method to assess the identification of pure samples of parental species and samples of mixed ancestry. Such tools, provided by the STRUCTURE and NEWHYBRIDS software) are advantageous because the analyses are able to indicate both contemporary hybridization and events of past hybridization, detecting hybrid categories going back to two or three generations (Gilabert and Wasmuth, 2013). Thus, future work using highly polymorphic markers, such as microsatellites, SNPs, and model-based Bayesian clustering methods, will enable us not only to clarify other hybridization events between closely related parasite taxa, but also to provide knowledge on the ecological significance and phenotypic characteristics of hybrid parasites.

## Supplementary material

For supplementary material accompanying this paper visit http://dx.doi.org/10.1017/S0031182016000330. click here to view supplementary material
