# Supplementary figures and images for: No more time to stay ‘single’ in the detection of Anisakis pegreffii, A. simplex (s. s.) and hybridization events between them: a multi-marker nuclear genotyping approach
Source: Parasitology. 2016 Apr 5;143(8):998–1011. doi: 10.1017/S0031182016000330 (PMC4890558; doi:10.1017/S0031182016000330)

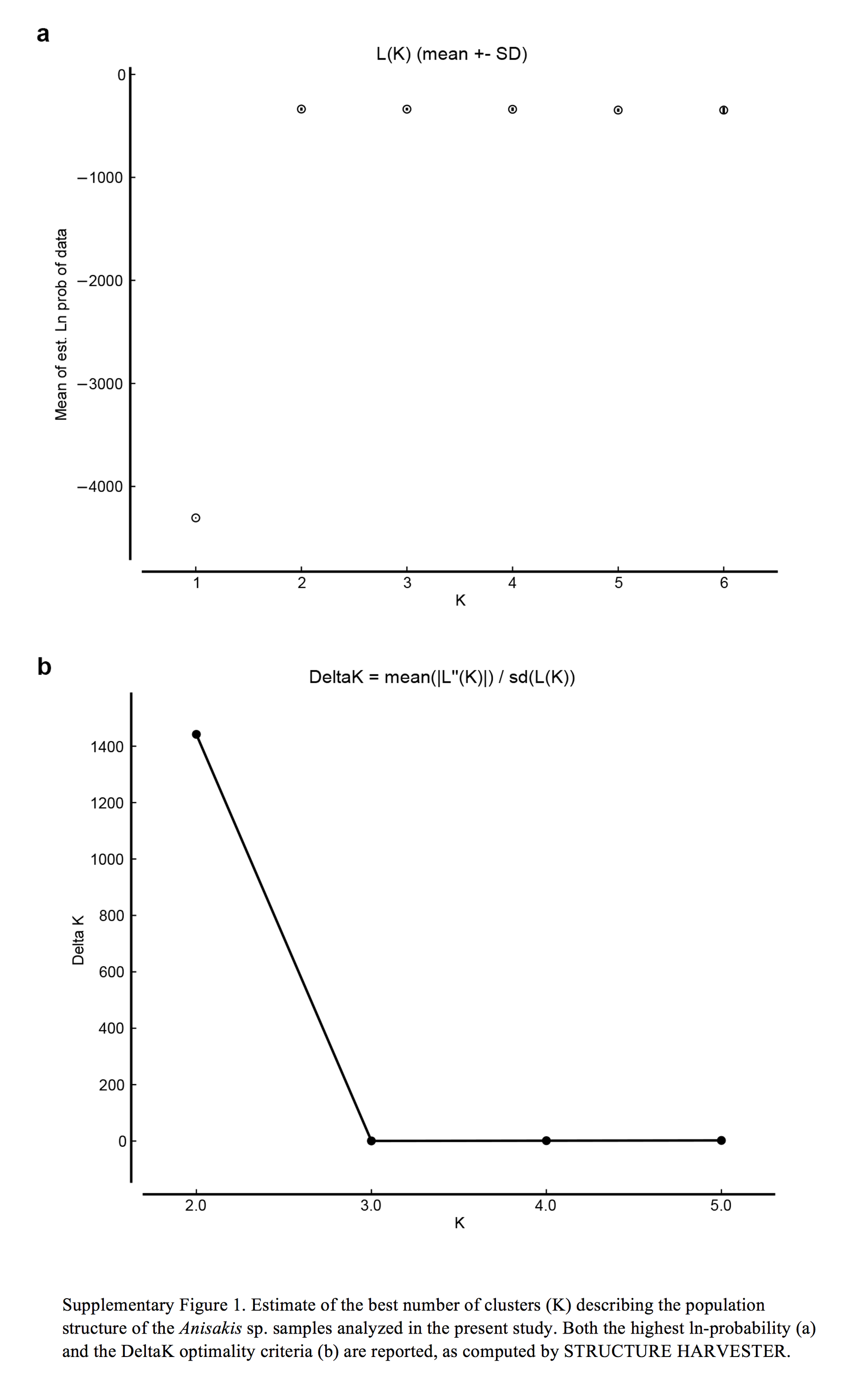

Supplement: Supplementary file 1 [file S0031182016000330sup001.tif]
